# Binge Eating, But Not Other Disordered Eating Symptoms, Is a Significant Contributor of Binge Drinking Severity: Findings from a Cross-Sectional Study among French Students

**DOI:** 10.3389/fpsyg.2017.01878

**Published:** 2017-10-30

**Authors:** Benjamin Rolland, Mickael Naassila, Céline Duffau, Hakim Houchi, Fabien Gierski, Judith André

**Affiliations:** ^1^Groupe de Recherche sur l’Alcool & les Pharmacodépendances (GRAP), INSERM ERi 24, Centre Universitaire de Recherche en Santé, Université de Picardie Jules Verne, Amiens, France; ^2^C2S Laboratory (EA 6291), University of Reims Champagne-Ardenne, Reims, France

**Keywords:** binge drinking, binge eating, binge-eating disorder, alcohol drinking, adolescent

## Abstract

Many studies have suggested the co-occurrence of eating disorders and alcohol use disorders but in which extent binge eating (BE) and other disordered eating symptoms (DES) are associated with the severity of binge drinking (BD) remains unknown. We conducted a online cross-sectional study among 1,872 French students. Participants were asked their age, gender, tobacco and cannabis use status. They completed the Alcohol Use Questionnaire (AUQ), Eating Disorder Examination Questionnaire (EDE-Q), and UPPS impulsive behavior questionnaire. BD score was calculated using the AUQ. Three items of the EDE-Q were used to construct a BE score. The predictors of the BD score were determined using a linear regression model. Our results showed that the BE score was correlated with the BD score (β_0_ = 0.051 ± 0.022; *p* = 0.019), but no other DES was associated with BD, including purging behaviors. The severity of BD was also correlated with younger age, male gender, tobacco and cannabis use, and with the ‘positive urgency,’ ‘premeditation,’ and ‘sensation seeking’ UPPS subscores (*R*^2^ of the model: 25%). Within DES, BE appeared as an independent determinant of the BD severity. This is in line with the recent hypothesis that BE is not a subtype of DES, but more a general vulnerability factor of emotional dysregulation, which could be shared by different behavioral and addictive disorders.

## Introduction

Alcohol use disorders (AUDs) (alcohol abuse and addiction) often co-occur with eating disorders. Bulimia nervosa and bulimic behaviors, binge eating (BE), purging, anorexia nervosa and atypical eating disorders have been associated with AUDs in women in a meta-analysis study (Gadalla and Piran, 2007; Baker et al., 2010; Root et al., 2010).

Binge drinking (BD) consists of episodic heavy alcohol drinking, and is often associated with drunkenness-oriented alcohol use^1^. Similarly, BE has been defined in the DSM-5 as abnormal eating episodes, which comprise eating much more rapidly or much larger amounts of food than normal, eating alone because of being embarrassed by how much one is eating, and feeling disgusted with oneself, depressed, or guilty after overeating.

Common cognitive and behavioral features have been described, and common integrative models have been proposed, with regard to BD and BE (Ferriter and Ray, 2011). BD and BE may share several features, such as repetitive engagement in the behavior despite evidence negative consequences (physical problems and poor academic performance), personality correlate such as neuroticism, and affected characteristics such as elevated levels of negative affect (impulsivity, anxiety, and depression). Concerning the common explanatory models of BD and BE, existing research proposed several key models such as the basic functional model, the motivational model, the expectancies model and the craving model (Ferriter and Ray, 2011).

Nevertheless, the possible interrelationships between BD and BE have only started to be explored. A couple of previous studies found frequent prevalence association between BE and BD, especially in women (Luce et al., 2007; Khaylis et al., 2009). However, though a characterized BE disorder has been defined by the DSM-5, BD remains a very heterogeneous set of drinking behaviors, with multiple and sometimes-questioned official definitions (Courtney and Polich, 2009). In many epidemiological studies, BD is frequently investigated by delineating populations using a cut-off drinking threshold (Courtney and Polich, 2009), which mixes BD subjects into a same group, and makes hard to address severity factors. For example, according to the World Health Organization (WHO), BD is defined as consuming at least 60 g of alcohol per drinking episode but some subjects drink at levels far beyond this binge threshold making difficult the study of severity aspects. That is why some authors have proposed using BD severity scores (Townshend and Duka, 2005). The BD score founded on patterns of drinking, rather than only quantities of alcohol consumed, may be more relevant of BD behavior.

In a more than 1800-subject sample of French students, we scored both BE and BD, as well as other DES, and we analyzed in which extent the BD score was determined by the scores of BE and other DES. It has never been assessed whether BE, as well as other disordered eating symptoms (DES), were associated with BD, not in terms of co-occurrence frequency, but as a specific severity factor of BD. In addition, since impulsivity has been consistently linked to the development and expression of BE and BD behaviors we assessed impulsivity behavior that may represent a common vulnerability factor.

## Materials and Methods

### Study Design and Participants

The study was an online anonymous survey conducted among all the students attending the French University of Rennes 1 in year 2012. 29,000 Students were invited to complete the questionnaire via their individual university email address. Students of Rennes 1 university are distributed as follows: 39% law, economy, management and human sciences, 28% health, 33% sciences, engineering and technologies. The link into the study could be activated only once, to avoid multiple participations in the survey. The identity of the participants completing the anonymous questionnaire was unknown to the researcher. No written informed consent was asked to the participants and the researcher’s contact information was indicated in the questionnaire. Students were able to continue with the survey only if they stated that they do consent to participate by ticking the consent button after reading the consent form (purpose of research, participation, procedure, confidentiality, and researcher’s contact information). Raw data were stored on a computer not connected to an internet network and were destroyed at the end of the study. The protocol was approved by the regional ethics committee (Comité de Protection des Personnes Nord-Ouest II).

### Questionnaire and Type of Data Collected

Participants were asked to provide their age, gender, weight, height, current tobacco smoking status, and current cannabis use status. They were also invited to complete online versions of the Alcohol Use Questionnaire (AUQ) (Mehrabian and Russell, 1978), the Eating Disorder Examination Questionnaire (EDE-Q) (Fairburn and Beglin, 1994), and the 20-item Urgency – Premeditation – Perseverance – Sensation seeking (UPPS) impulsive behavior questionnaire (Billieux et al., 2012).

### Score Construction

A BD score was calculated on the basis of three items of the AUQ, as previously validated (Townshend and Duka, 2005). The BD score was calculated for all participants on the basis of the information given in items 10, 11, and 12 of the AUQ [Speed of drinking (average drinks per hour); number of times being drunk in the previous 6 months; percentage of times getting drunk when drinking (average)]. The BD score is calculated as follows [4 × (Item 10) + Item 11 + 0.2 × (Item 12)]. This score gives a picture of the drinking patterns of the participants rather than just a measure of alcohol intake. Using the UPPS questionnaire, scores of ‘negative urgency,’ ‘positive urgency,’ ‘lack of premeditation,’ ‘lack of perseverance,’ and ‘sensation seeking’ were calculated for each participant (Billieux et al., 2012). Moreover, the Body Mass Index (BMI) of respondents was calculated on the basis of their reported weight and height. Using the EDE-Q questionnaire, scores of ‘dietary restraint,’ ‘eating concern,’ ‘shape concern,’ and ‘weight concern’ were calculated as defined by the authors of the questionnaire (Fairburn and Beglin, 1994). In addition, a BE score was calculated by summing the subscores of the items 13, 14, and 15, of the EDE-Q, while a ‘purging behaviors’ score was obtained by summing the specific questions of the EDE-Q, i.e., questions 16 and 17. Moreover, categorical BMI groups were constructed. All the subjects with a BMI of less than 18.5 were defined as the ‘underweight’ group. The ‘≥18.5 to <25’ group was defined as ‘normal,’ while the ‘≥25 group’ was defined as the ‘overweight’ group.

### Statistical Analysis

Categorical variables are provided as the number and percentage (n; %). Quantitative variables are provided as the mean and standard deviation, and median and interquartile range (mean ± SD; med [IQR]). For both the BD and BE scores, bivariate analyses were conducted to explore the association with the other parameters explored. Comparisons between two quantitative measures were performed using the Spearman’s ρ test, whereas comparisons between quantitative and categorical variables were performed using Mann–Whitney or Kruskal–Wallis tests.

Furthermore, a multivariable linear regression modeling was built, with the BD score as the dependent variable, and other variables as the explanatory variables. The standardized coefficients of the model are provided with their standard deviation (β_0_ value ± SD). The significance threshold was fixed at 0.05 for all tests. Analyses were conducted using the XLSTAT2014 software^2^.

## Results

Of the 29,000 students who were invited to complete the online questionnaire, 1,872 accepted to participate (mean age = 21.1 ± 2.44 years; median age = 21 [20–23]); 57.4% females; 21.4% tobacco smokers; 29.6% cannabis users).

Results of the bivariate comparisons of the BD and BE scores with other variables provided in the **Table 1**.

**Table 1:** Bivariable comparisons for the BE and BD scores.

	BE score	*p*-value	BD score	*p*-value
Age	ρ = -0.03	0.28	ρ = -0.14	<0.0001
Females (vs. males)	0 [0–3] vs. 1 [0.2]	0.9	11 [6–21] vs. 17 [8–30]	<0.0001
Tobacco smokers (vs. non-smokers)	1 [0–3] vs. 1 [0–2]	0.025	21 [12–36] vs. 11 [4–21]	<0.0001
Cannabis users (vs. non-users)	1 [0–3] vs. 0 [0–2]	0.049	23 [14–36] vs. 10 [4–19]	<0.0001
BD score	ρ = 0.12	<0.0001	–	–
BE score	–	–	ρ = 0.12	<0.0001
EDE-Q – purging behaviors	ρ = 0.17	<0.0001	ρ = 0.03	0.18
EDE-Q – dietary restraint	ρ = 0.19	<0.0001	ρ = 0.03	0.18
EDE-Q – eating concern	ρ = 0.36	<0.0001	ρ = 0.006	0.78
EDE-Q – shape concern	ρ = 0.28	<0.0001	ρ = 0.012	0.60
EDE-Q – weigh concern	ρ = 0.26	<0.0001	ρ = 0.009	0.70
BMI (quantitative)	ρ = 0.17	<0.0001	ρ = 0.02	0.38
BMI ([<18.5] vs. [18.5–25])	0 [0–1] vs. 1 [0–2]	0.095	12 [4–22] vs. 14 [7–24]	0.11
BMI ([>25) vs. [18.5–25])	2 [0–3] vs. 1 [0–2]	0.002	12 [6–17] vs. 14 [7–24]	0.11
UPPS – negative urgency	ρ = 0.18	<0.0001	ρ = 0.09	<0.0001
UPPS – positive urgency	ρ = 0.21	<0.0001	ρ = 0.22	<0.0001
UPPS – lack of premeditation	ρ = 0.08	0.001	ρ = 0.21	<0.0001
UPPS – lack of perseverance	ρ = 0.06	0.007	ρ = 0.13	<0.0001
UPPS – sensation seeking	ρ = 0.08	0.001	ρ = 0.22	<0.0001

*Comparisons between quantitative variables were performed using the Spearman’s correlation test (ρ). Comparisons between categorical and quantitative variables were conducted using the Mann–Whitney test, and are provided as the median and the interquartile range (med [IQR]). BD, binge drinking; BE, binge eating; EDE-Q, Eating Disorder Examination Questionnaire; UPPS, ‘urgency – premeditation – perseverance – sensation seeking’ impulsive behavior scale.*

The BD score was significantly associated with the BE score (ρ = 0.12; *p* < 0.0001), but not with other EDE-Q subscores. The BD score was also significantly associated with male gender (*p* < 0.0001), tobacco smoking status (*p* < 0.0001), and cannabis use status (*p* < 0.0001). It was also significantly correlated with every UPPS subscore (*p* < 0.0001 for each), and negatively associated with age (ρ = -0.14; *p* < 0.0001). Consequently, all these parameters were integrated in the multivariable linear regression model, which is provided in the **Table 2**.

**Table 2:** Results of the multivariable linear regression modeling of the BD score (β_0_ = normalized coefficients; *R*^2^ = 25%).

	β_0-_value ± SD	*p*-value
Age	-0.104 ± 0.021	<0.0001
Female gender	-0.175 ± 0.023	<0.0001
Tobacco smoker	0.129 ± 0.022	<0.0001
Cannabis user	0.257 ± 0.023	<0.0001
BE score	0.051 ± 0.022	0.019
EDE-Q – purging behaviors	-0.009 ± 0.021	0.680
EDE-Q – dietary restraint	0.042 ± 0.028	0.140
EDE-Q – eating concern	-0.034 ± 0.031	0.270
EDE-Q – shape concern	0.010 ± 0.055	0.857
EDE-Q – weigh concern	0.032 ± 0.055	0.565
Body mass index	-0.042 ± 0.023	0.069
UPPS – Negative Urgency	-0.016 ± 0.024	0.494
UPPS – positive urgency	0.104 ± 0.025	<0.0001
UPPS – lack of premeditation	0.105 ± 0.024	<0.0001
UPPS – lack of perseverance	0.023 ± 0.023	0.328
UPPS – sensation seeking	0.064 ± 0.022	0.004

*BD, binge drinking; BE, binge eating; CI95%, confidence interval 95%; EDE-Q, Eating Disorder Examination Questionnaire; SD, standard deviation; UPPS, ‘urgency – premeditation – perseverance – sensation seeking’ impulsive behavior scale.*

In the multivariable modeling, the BE score remained significantly correlated with the BD score (β_0-_value = 0.051 ± 0.022; *p* = 0.019), whereas the scores of other DES were not significantly associated with the BD score. Male gender, younger age, tobacco smoking status, and cannabis use status, were all significant contributors of the BD score (*p* < 0.0001 for each parameter). In addition, only the ‘positive urgency’ (*p* > 0.0001), ‘lack of perseverance’ (*p* < 0.0001), and ‘sensation seeking’ (*p* = 0.004) subscores of the UPPS scale were significantly associated with the BD score. The overall goodness of fit of the model was *R*^2^ = 25%.

## Discussion

The main objective of the study was to assess in which extent the different DES were significant contributors of the severity of BD in a population of French students. In this respect, we found that only the severity of BE, and not other dimensions of disordered eating, was significantly correlated to the BD score. Moreover, the BE score appeared also significantly correlated with all other EDE-Q subscores and with the BMI, whereas these different quantitative parameters were not correlated with the BD score.

These findings are consistent with some recent hypotheses, according to which BE should not be viewed as a subcategory of DES, but as the more general expression of an impaired emotion regulation, which would constitute a common vulnerability factor for eating disorders, as well as other addictive behaviors (Stojek et al., 2014; Leehr et al., 2015; Eichen et al., 2016). Impulsivity has been regularly, though inconstantly, associated with this BE-related emotional dysregulation (Schag et al., 2013; Stojek et al., 2014; Eichen et al., 2016). Consequently, it was important to adjust our analyses using impulsivity traits assessment to explore the severity of BD. However, this did not change our main results. Moreover, differentiating between BE and purging behaviors was never previously addressed in previous research on eating behaviors. In this regard, we found that purging behaviors, though highly associated with BE, were not associated with BD. To our knowledge, this is a second original finding.

Furthermore, our results are in line with several previous findings. ‘Positive urgency’ and ‘sensation seeking’ were both associated with substance use (Billieux et al., 2012), whereas ‘negative urgency’ was more associated with substance dependence (Verdejo-García et al., 2007). In our study, which did not focus on dependent subjects, we found a significant association between the BD score and the ‘positive urgency’ and ‘sensation seeking’ subscores of the UPPS. Moreover, previous investigations reported an association between BE and either ‘negative urgency’ (Bardone-Cone et al., 2016), or both ‘negative’ and ‘positive’ urgency dimensions (Stojek et al., 2014). In our study, we confirmed these associations, as, among all the UPPS subscores, both urgency subscores were those which showed the strongest association with the BE score.

Several limitations should also be acknowledged regarding this study. First and foremost, the response rate was only about 6.4% and can be partially explained by the fact that the majority of students do not use the email address provided by the university [but is not very low compared to that of similar studies (Tavolacci et al., 2016)]. We cannot exclude that the more involved or problematic individuals refused to participate and the lack of psychiatric interview is also an important limitation here since we did not detect the presence of psychiatric diagnosis in our sample. The entirely self-report dimension of the data analyzed may have impacted the reliability of the data despite the large sample size recruited and it has been already shown that for alcohol consumption, it may be underestimated by the use of retrospective questionnaire (Townshend and Duka, 2002), the self-report ascertainment of cannabis use may also be affected by the fact that some cannabis users may deny using cannabis.

The building of the BD score followed a validated procedure (Townshend and Duka, 2005) while the way the BE and purging scores were constructed was not based on previous studies. The global EDE-Q score has shown good psychometric properties to measure BE (Vander Wal et al., 2011), but no specific study has ever demonstrated the validity of the items we selected to, respectively, score the BE and purging behaviors. However, these items specifically focus on BE or purging symptoms. It is noteworthy that ‘purging behavior’ is not only defined by vomiting and laxative misuse (as highlighted in the EDE-Q) but also by other behaviors such as misuse of diuretics, infusions and sugar-free candies. Another possible limitation of the study is that no association was found between the female gender and the BE score, whereas BE is usually much more frequent among females (Allen et al., 2014). However, in the present study, we did not use a frequency but a severity assessment, which is not similar. The lack of between-gender difference would be questionable if BE would have been more severe among women, and not only more frequent. To our knowledge, this has not been demonstrated yet.

## Conclusion

Overall, we found that the BE severity was correlated with the BD severity, contrary to other DES. These results suggest that BE could consist of a general vulnerability factor, underlying elements of emotional dysregulation that remain to be more understood. This common vulnerability could link different types of behaviors and mental disorders, which may elsewhere be poorly interrelated, like, in our study, BD and DES.

## Author Contributions

MN and JA developed the study. All authors contributed to the study design. Data collection was conducted by CD and JA and data analyses were performed in collaboration with all authors (BR, MN, CD, HH, FG, and JA). BR drafted the paper under the supervision of MN, while HH, FG, and JA provided critical revisions. All authors approved the final version of the paper.

## Conflict of Interest Statement

BR has provided expert testimony for Ethypharm and Indivior. And received lecture fees from Ethypharm, Lundbeck, Indivior, Bouchara-Recordati, Gilead, AstraZeneca, Bristol-Myers-Squibb, Otsuka, and Servier. MN received lecture or expert fees from Merck-Serono, Lundbeck and Bouchara-Recordati. Both BR and MN received grants from the Fondation Actions-Addictions. These funds did not exert any editorial direction or censorship on any part of this article. JA, HH and FG have no conflict of interest to declare regarding the present article. The other authors have no conflict of interest to declare regarding the present article.

## Footnotes

1 National Institute on Alcohol Abuse and Alcoholism (NIAAA). Drinking Levels Defined n.d. https://www.niaaa.nih.gov/alcohol-health/overview-alcohol-consumption/moderate-binge-drinking (accessed August 8, 2016).

2 https://www.xlstat.com/en/
